# Identifying Gaps in Obstetrics and Gynecology Education: Insights From Clinical Vignettes

**DOI:** 10.7759/cureus.96992

**Published:** 2025-11-16

**Authors:** Mia Ramirez, Ashley Shin, Elizabeth K Nugent

**Affiliations:** 1 Department of Obstetrics and Gynecology, Tulane School of Medicine, New Orleans, USA; 2 Division of Plastic and Reconstructive Surgery, Baylor Scott & White Medical Center - Temple, Temple, USA; 3 Department of Obstetrics, Gynecology and Reproductive Sciences, McGovern Medical School at UTHealth Houston, Houston, USA

**Keywords:** gynecology, lgbtq+ health, medical education, obstetrics, scope of practice, women’s health

## Abstract

Introduction

Obstetrics and gynecology (Ob/Gyn) is a diverse specialty encompassing primary care, surgical procedures, and multiple subspecialties. Medical students may have limited understanding of its scope, particularly as clinical practice and available subspecialties vary across institutions.

Methods

A cross-sectional survey consisting of 12 clinical vignettes was administered to medical students at UTHealth Houston McGovern Medical School. Respondents provided demographic data, indicated their specialty of interest, and reported prior exposure to Ob/Gyn. For each vignette, students selected the specialty they believed most appropriate for patient management.

Results

Seventy-four responses were analyzed (70.3% female, 29.7% male). Among participants, 21.6% expressed interest in pursuing Ob/Gyn, and 68.9% reported prior exposure. Ob/Gyn was most frequently selected for women’s gynecologic health screenings (66.2%), fertility planning (97.3%), and evaluation of vulvar lesions causing dyspareunia (87.8%). It was less frequently chosen for ovarian torsion (37.8%), urinary incontinence (35.1%), and LGBTQ+ health (6.8%).

Conclusion

Medical students demonstrated adequate understanding of Ob/Gyn’s role in fertility planning, Pap smear screening, and management of dyspareunia but showed knowledge gaps regarding gynecologic emergencies, urinary incontinence, gynecologic malignancy, and LGBTQ+ health. Early clinical mentorship, targeted didactic content, and simulation-based learning may help improve understanding of the scope of Ob/Gyn practice.

## Introduction

Obstetrics and gynecology (Ob/Gyn) is both a primary care and surgical field, with opportunities to subspecialize through fellowships. In addition to established subspecialties of Ob/Gyn, such as urogynecology and reconstructive pelvic surgery, gynecologic oncology, reproductive endocrinology and infertility, and maternal-fetal medicine, more recently designated subspecialties such as pediatric and adolescent gynecology, reproductive infectious disease, and complex family planning are emerging. It is not surprising that medical students may lack exposure to and knowledge of the broad scope of Ob/Gyn, especially as clinical practice and available subspecialties vary by institution [1]. With an ongoing projected need for more Ob/Gyn physicians in the United States, a better understanding of the varied roles of Ob/Gyns may spark medical student interest in the field, lead to more appropriate referrals, and improve patient outcomes with more expeditious treatment [2].

Because of the wide range of treatments provided by Ob/Gyns, as well as the various subspecialties within the field, this study evaluated how often students chose to primarily involve Ob/Gyn care across a variety of clinical scenarios. Any knowledge deficits identified should be addressed within the medical school curriculum to ensure that graduates have an adequate understanding of the role of Ob/Gyn in clinical practice. This study aims to identify and analyze gaps in medical students' understanding of the scope of Ob/Gyn by assessing their responses to clinical vignettes. Secondarily, this study evaluated how prior clinical exposure influences medical students’ perception of when Ob/Gyn provides care as the primary team.

Previous studies have queried medical students, healthcare professionals, and the public on their perceptions of specialties such as plastic surgery, vascular surgery, and oral and maxillofacial surgery [3-5]. In 2010, Farber et al. reported that although medical students had a better understanding of the broad scope of vascular surgery compared to the general public, there was still room for improvement [3]. The study also found that if a medical student personally knew a vascular surgeon, they demonstrated an improved understanding of the scope of vascular surgery. Furthermore, residency programs generally have competency-based medical education requirements, which may not reflect true daily practice [6]. If students and residents do not have a good understanding of sexual health practices or providers who specialize in these aspects of women’s health, patients may experience delayed referrals to the appropriate practitioners or receive suboptimal care [7].

## Materials and methods

Study design

This survey study, titled Evaluating the Medical Student Perception of the Scope of Obstetrics and Gynecology, was approved for expedited review by the University of Texas Health Science Center at Houston Institutional Review Board (HSC-MS-24-0530). A 17-item survey consisting of five demographic questions and 12 clinical scenarios was administered to medical students at UTHealth Houston McGovern Medical School (McGovern Medical School). Participants provided basic demographic information (gender and age), indicated their specialty of interest, and reported prior exposure to Ob/Gyn. For each vignette, students selected a preferred specialty from a multiple-choice list to provide patient care, with the objective of identifying in which scenarios students selected Ob/Gyn, regardless of perceived correctness. 

Answer choices included specialties with overlapping clinical purview (e.g., Family Medicine) to better assess students’ perceptions of the role of Ob/Gyn relative to other fields. The answer choices purposely included specialties to mimic the ambiguity of real-life scenarios. The survey was converted into an anonymous, web-based format using Qualtrics software (Qualtrics, Provo, UT) and distributed along with an informed consent disclosure statement via email to all students enrolled at McGovern Medical School in Houston, Texas, between June and August 2024. Responses were also solicited at the start of third-year Ob/Gyn clinical clerkship lectures, as this class had the lowest percentage of respondents. Surveys completed by students aged 18 years or older who were accepted and enrolled at McGovern Medical School during the study period were included. No actively enrolled medical students were excluded from participation. Incomplete surveys and responses submitted outside the designated study period were excluded during data analysis. No incentives or reminders were sent via email to encourage survey completion.

Clinical experience was defined as officially mandated medical school clerkship participation. Non-clinical experience was defined as exposure to healthcare through indirect activities such as academic research or physician shadowing. The category “No Clinical Experience” included participants who reported no exposure to Ob/Gyn topics in either a clinical or non-clinical context. The category “No Ob/Gyn Interest” included participants who expressed interest in any specialty other than Ob/Gyn.

Study instrument

A structured questionnaire was developed by the authors to evaluate medical student knowledge of Ob/Gyn topics. The instrument consisted of two parts. The first section collected demographic information including age, gender, class year, and prior exposure to Ob/Gyn (e.g., shadowing, preceptorship, clinical rotation, or none). Demographic information was obtained through multiple-choice or free-response answers. The second section contained a series of clinical vignettes designed to assess knowledge of Ob/Gyn diagnoses and decision-making in consultation scenarios.

Clinical topics related to Ob/Gyn included women’s gynecologic health screening, fertility management, gynecologic emergencies, obstetric surgical complications, urinary incontinence, dyspareunia, gynecologic oncology, and LGBTQ+ health, as these are relevant within the field. Additional topics, pneumonia, acne vulgaris, skin malignancy, and chronic musculoskeletal pain, were included to represent conditions typically managed by other specialties. Ob/Gyn-related vignettes were designed with attention to current Ob/Gyn subspecialty training opportunities as outlined by the National Resident Matching Program (complex family planning, gynecologic oncology, maternal-fetal medicine, minimally invasive gynecologic surgery, pediatric and adolescent gynecology, and reproductive endocrinology), high-yield diagnoses, and community practice standards.

Multiple-choice answer options were structured to include relevant specialties (e.g., Family Medicine, Internal Medicine, General Surgery, Urology, Ob/Gyn). Final vignette selection and content validity were established through expert review by the senior author within the Department of Obstetrics and Gynecology at McGovern Medical School. The survey was administered in its entirety to all participants without modification.

The full questionnaire is provided in Appendix A to ensure transparency and reproducibility.

Data analysis

Demographic data were analyzed using descriptive statistics. Due to the limited selection of some multiple-choice answers, Fisher’s exact tests were performed in RStudio (version 3.4.1; RStudio, Boston, MA) to compare bifurcated categorical demographic variables with the selection of Ob/Gyn as the preferred specialty for each vignette. Statistical significance was defined as p < 0.05. For vignettes demonstrating statistically significant or near-significant results, a one-tailed Fisher’s exact test was applied to assess directionality.

## Results

**Figure 1:**
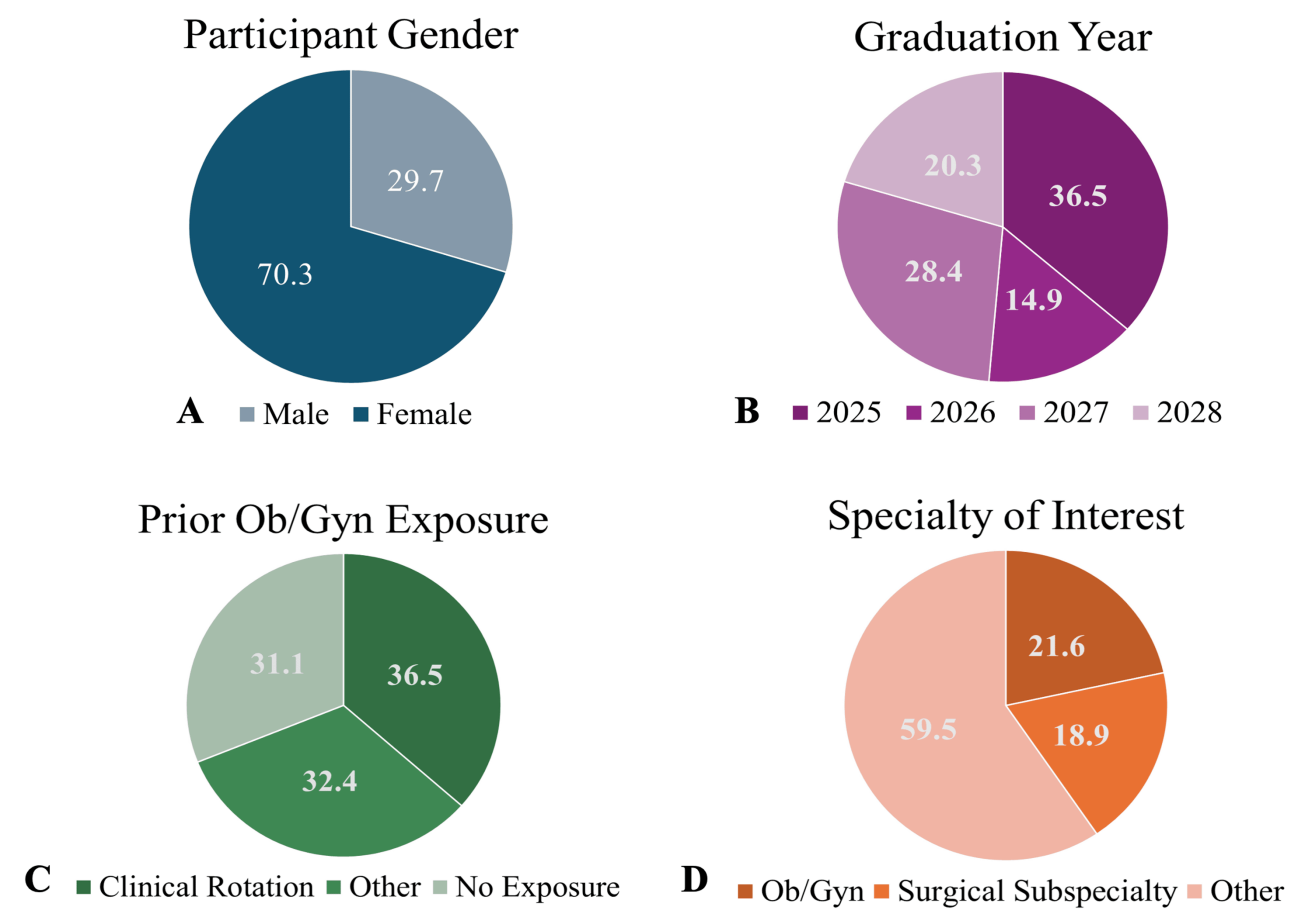
Distribution of participant demographic information obtained from the survey, including gender (A), graduation year (B), prior exposure to Ob/Gyn (C), and specialty of interest (D) at the time of the study. Demographic data were analyzed using descriptive statistics.

Among the 74 respondents (70.3% female, 29.7% male), 21.6% expressed interest in pursuing Ob/Gyn. A total of 68.9% reported clerkship, shadowing, or research experience in Ob/Gyn. Students from all four years of medical school participated in the survey. Demographic data are presented in Figure 1.

**Figure 2:**
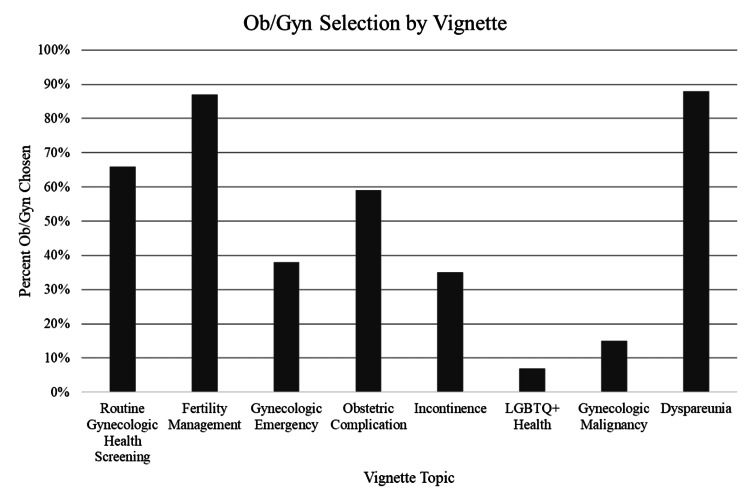
Eight vignettes on women’s health-related problems were included in the survey The figure displays the percentage of students who selected Ob/Gyn for each women’s health-related problem.

Ob/Gyn care was most frequently selected for women’s gynecologic health screenings (66.2%), fertility planning (97.3%), and vulvar lesions causing dyspareunia (87.8%). It was least frequently chosen for ovarian torsion (37.8%), urinary incontinence (35.1%), and LGBTQ+ health (6.8%) (Figure 2).

As students gained more clinical training, they were more likely to select Ob/Gyn for the management of ovarian torsion (fourth year: 70.4% vs. third year: 54.6%) and less likely to select Ob/Gyn for annual health screenings (fourth year: 55.6% vs. first year: 80.0%). For routine gynecologic health screening, students selected appropriate care, with 66.2% choosing Ob/Gyn and 33.8% selecting either Family Medicine or Internal Medicine. Responses regarding urinary incontinence and selecting Ob/Gyn care varied by year: fourth years (44.4%), third years (36.4%), second years (14.3%), and first years (47.0%). Only 18.5% of fourth-year students, and none of the students with less clinical experience, selected Ob/Gyn for providing LGBTQ+ healthcare.

When comparing students with and without prior clinical exposure to Ob/Gyn, those who had such exposure were significantly more likely to identify Ob/Gyn as the appropriate specialty for managing gynecologic emergencies, LGBTQ+ health concerns, and gynecologic malignancies (two-tailed Fisher’s exact test, p < 0.01 for all). Additionally, participants with clinical exposure demonstrated a consistent tendency to select Ob/Gyn for the management of obstetric complications, urinary incontinence, pneumonia, and dyspareunia or other benign gynecologic conditions, although these differences did not reach statistical significance. 

When exploratory sub-analysis was performed, differentiating clinical exposure to Ob/Gyn from volunteer or non-academic exposure (n = 51), Ob/Gyn providers were selected more frequently for managing gynecologic emergencies, obstetric complications, and gynecologic malignancy when students had experienced clinical education in Ob/Gyn (p < 0.01, p < 0.05, and p < 0.01, respectively). Similarly, clinical exposure showed a trend toward increased selection of Ob/Gyn providers for treating LGBTQ+ health concerns and urinary incontinence (Table 1).

**Table 1:** The impact of clinical experience versus preclinical experience (including no experience) and versus other non-clinical experience (excluding no experience) on the selection of Ob/Gyn for each vignette topic Fisher’s exact tests were performed using RStudio (version 3.4.1; RStudio, Boston, MA) to evaluate the impact of clinical experience versus preclinical experience (including no experience) and versus other non-clinical experience (excluding no experience) on the selection of Ob/Gyn for each vignette topic. Statistical significance was defined as p < 0.05. An asterisk (*) indicates a statistically significant p-value.

Vignette Topic	Clinical vs. preclinical experience (including no experience) n = 74	Clinical vs. other non-clinical experience (excluding no experience) n = 51
Routine Gynecologic Health	0.45	0.37
Fertility Management	0.53	0.22
Gynecologic Emergency	< 0.01*	< 0.01*
Obstetric Complication	0.08	< 0.05*
Incontinence	0.09	0.15
Pneumonia	0.08	0.11
Acne Vulgaris	1	1
LGBTQ+ Health	< 0.01*	0.05
Gynecologic Malignancy	< 0.01*	< 0.01*
Musculoskeletal Pain	1	0.72
Dyspareunia and Benign Gynecology	0.07	0.26
Skin Malignancy	0.37	1

Participant gender and self-reported interest in Ob/Gyn were not significantly associated with selecting Ob/Gyn for any clinical vignette.

## Discussion

Participants in our single-institution study demonstrated a generally high level of familiarity with Ob/Gyn, though comparatively few indicated an interest in entering the specialty. Ob/Gyn care was most associated with routine screenings, fertility concerns, and the management of conditions affecting sexual comfort. In contrast, participants were less likely to view Ob/Gyn as the primary specialty for acute gynecologic emergencies, pelvic floor disorders, or care tailored to the LGBTQ+ community. These patterns suggest that while core aspects of women’s health are widely recognized as central to Ob/Gyn practice, the broader and more specialized scope of the field may be underappreciated. Participants who reported greater clinical exposure to the field more frequently selected Ob/Gyn care in these types of patient scenarios 

Although fourth-year medical students were less likely to indicate Ob/Gyn care for women’s annual health screenings, this may reflect clinical exposure to routine women’s health services in other primary care settings, such as Family Medicine or Internal Medicine. Given that the scope of women’s health services varies widely among providers, students may have been able to distinguish between basic gynecologic health screening and cases more appropriately referred for specialized gynecology appointments.

Ob/Gyn emergencies and surgical complications

Despite the benefits of our large academic institution, including high patient care volume and complex care coordination, this study highlights areas where students may lack critical knowledge for providing optimal patient care. Topics such as ovarian torsion, urinary incontinence, and LGBTQ+ health require greater emphasis in the curriculum so that students can develop confidence in managing these aspects of care. Ob/Gyn emergencies, as well as surgical complications, are particularly important for students to understand because patient safety is a priority for all future providers. Although ovarian torsion is a relatively rare diagnosis, timely recognition is essential, as surgical delays can lead to ovarian loss and future infertility [8]. Our findings demonstrate that students with prior clinical experience were more likely to select Ob/Gyn providers for the management of ovarian torsion, underscoring the importance of emphasizing this material during required clinical education, such as the Ob/Gyn clerkship, rather than leaving it to optional clinical exposures.

To better integrate clinically relevant information into medical education, the risks, benefits, and alternatives of surgical procedures should be regularly discussed when students assist in routine operations. For example, ureteral injuries during cesarean section are uncommon but can be difficult to identify intraoperatively [9]. In our clinical vignette depicting an inability to spontaneously void after cesarean section, only 59% of participants selected Ob/Gyn as the appropriate provider for this obstetric surgical complication. However, clinical experience was statistically significantly associated with selecting Ob/Gyn for this postoperative complication. Incorporating discussions of surgical complications into clinical rotations may improve students’ ability to recognize signs of a surgical abdomen and manage complications of procedures in which they participate, regardless of the specialty they ultimately pursue.

Another strategy to enhance students’ clinical training is the integration of simulation-based learning into undergraduate medical education [10]. In 2019, Carpenter and Rowlands reported that simulation-based obstetric emergency education improved consolidation of knowledge, enhanced non-technical skills, and increased confidence in applying these skills in real clinical situations among fourth-year medical students [11]. Offering preclinical students opportunities to evaluate simulated patients presenting with symptoms of ovarian torsion or ureteral injury may help them recall and apply high-yield diagnoses earlier in their medical careers. 

Urinary incontinence

Urinary incontinence is a common and often debilitating condition, affecting approximately 60% of Americans [12]. Despite its high prevalence and the likelihood of frequent clinical encounters, fewer than 40% of participants in our study selected Ob/Gyn care for managing urinary incontinence in women. This finding may be explained by several factors. Multiple specialties treat urinary incontinence; both Urology and Ob/Gyn providers can obtain subspecialty board certification in the management of female urinary incontinence, and treatment approaches may involve complex combinations of medical and surgical interventions [13]. Additionally, because of the social stigma associated with this condition, patients may be more likely to discuss it with their primary care providers. As a result, medical students, particularly those planning to enter primary care, may be the first or only clinicians to whom patients disclose these concerns [14]. Emphasizing recognition, early management, and appropriate referral of urinary incontinence in both preclinical and clinical education is essential to improving quality of life.

Sexual health

Sexual health-related issues, as defined by the World Health Organization, encompass sexual orientation and gender identity, sexual expression, relationships, and pleasure [15]. In our study, Ob/Gyn providers were rarely chosen as the primary care team for scenarios involving designated LGBTQ+ care. The Ob/Gyn clerkship may represent one of the few opportunities for students to receive structured education in sexual health; if this exposure is limited or de-emphasized, it may reduce future physicians’ confidence in providing appropriate care for diverse patient populations [7]. Increased curricular emphasis on general sexual health-related issues, many of which overlap with LGBTQ+ health, may better equip students to provide comprehensive and personalized care. Routine Ob/Gyn encounters also provide opportunities for providers to address sensitive topics such as sexual health, gender identity, and related concerns. Education on routine screening for intimate partner violence, which affects 15-71% of women over their lifetime, as well as awareness of human trafficking, elder abuse, and child abuse, can also be incorporated into this critical educational period [16]. Beyond didactics, students who have the opportunity to hear personal stories from patients and volunteer for organizations that support women may enhance learning and promote the understanding of a physician’s social responsibility within the community.

Strengths and limitations

This study is subject to several limitations. Single-institution sampling limits the diversity of the participant pool. The modest response rate (74 responses out of 1,086 possible respondents) introduces statistical limitations and may affect the robustness of the findings, and no a priori power calculation was performed, which restricts the ability to draw definitive conclusions or detect smaller effect trends. The level of clinical exposure was self-reported, which may be influenced by recall inaccuracies or social desirability bias, and could affect the precision of the findings. The possibility of response bias must be acknowledged, as those with greater interest in Ob/Gyn may have been more likely to participate, introducing selection bias and limiting generalizability to all U.S. medical students. Although our study did not find a significant association with specialty interest and choosing Ob/Gyn, students who express greater interest in Ob/Gyn may be more likely to seek out Ob/Gyn educational experiences, which would enhance their baseline understanding of the field. Finally, these results should not be overgeneralized to medical education systems outside the United States or to institutions with substantially different curricular structures, as training environments and exposure opportunities may vary considerably.

Additionally, the survey instrument used in this study was not psychometrically validated, which may limit the reliability and interpretability of the measured constructs. The inherent ambiguity within the overlapping responsibilities and scopes of practice between various specialties present in medical practice was purposely emulated in the survey and therefore could have elicited confusion for medical students. Although efforts were made to minimize wording bias during the creation of clinical vignettes, it is possible that phrasing influenced students’ responses. 

Strengths of this study include surveying students at a busy academic institution with multiple clinical sites and a diverse range of clinical experiences. The inclusion of both preclinical and clinical students, as well as vignettes spanning a variety of specialties, enhances the breadth of insights obtained.

Recommendations and future directions

Medical training experiences, particularly during the Ob/Gyn clerkship, vary considerably. Students’ exposure to sexual health and women’s health topics may be influenced by factors such as student gender, quality of feedback, mentorship or preceptorship, hospital and clinic settings, patient populations served, and overall organization of the rotation [17]. Given this inherent variability, consistent didactic instruction focused on emergent and common Ob/Gyn diagnoses, early management strategies with appropriate referrals to subspecialists, and general sexual health-related issues may help students achieve a more uniform and comprehensive understanding of the scope of Ob/Gyn practice. Active participation in evaluating a patient presenting with symptoms of ovarian torsion or postoperative complications, even through simulation, may help students to form lasting memories of the most high-yield diagnoses. 

As greater clinical exposure was associated with more frequent selection of Ob/Gyn care in this study, students may benefit from earlier integration of interdisciplinary patient care and clinical scenarios during the preclinical phase of medical education. Studies have shown that early clinical exposure helps students translate theoretical learning into decision-making and reasoning skills in clinical settings, which then enhances medical students’ motivation and reduces stress, and reinforces professional ethics [18-20]. Early clinical involvement aligns medical training with the health needs of the community and introduces students to the inherent ambiguity of real patient care, where decisions are seldom straightforward like in standardized examinations, thereby fostering the adaptive reasoning required in future practice [21].

Early clinical exposure and community-based scholarly activities, such as volunteering at a free women’s health clinic, could be beneficial for students to foster a sense of responsibility and connection within the community, especially in navigating situations of intimate partner violence and LGBTQ+ care. Increased opportunities for clinical exposure could be especially useful at institutions where a simulation lab or standardized patients are not readily available [10]. Understanding that students have a strong grasp on the basics of Ob/Gyn care, additions to the curriculum to include the wider breadth of Ob/Gyn could be helpful for students. 

As for the importance of mentorship, Rachoin et al. in 2023 reported that medical students are more likely to keep the same specialty preference over the course of medical school if they had positive mentorship within that specialty [22]. With the increasing need for Ob/Gyns and in a field where subspecialization after fellowship is common, Ob/Gyn mentorship should be prioritized for medical students to understand and experience the scope of the field before and while choosing their career path. 

Future research should explore barriers to recognizing Ob/Gyn-related conditions, assess the impact of early clinical exposure on students’ decision-making, and evaluate the potential benefits of incorporating simulation-based training into preclinical education. Modifications to this survey to include more nuanced aspects of Ob/Gyn care and various presentations of the same clinical diagnosis across various patient populations with greater detail. A future study across multiple institutions could assess how varying clinical exposures impact students' understanding of Ob/Gyn, which would improve external validity. Additionally, implementing longitudinal studies to assess how Ob/Gyn knowledge evolves throughout medical training could provide valuable insights into the effectiveness of curriculum interventions. Furthermore, studies could evaluate how Ob/Gyn education in medical school impacts competency at a residency level. 

## Conclusions

Within our single-institution study, medical student knowledge of Ob/Gyn care was consistently chosen for fertility planning and the management of dyspareunia. However, potential gaps remain in understanding the role of Ob/Gyn providers in managing surgical emergencies, urinary incontinence, gynecologic malignancies, and LGBTQ+ health. Addressing these gaps in understanding the scope of Ob/Gyn care could better equip medical students, support more sound clinical judgment, and ultimately contribute to improved patient care. This may require earlier clinical mentorship, targeted didactic content, and incorporation of additional learning opportunities, such as simulation-based training, within both preclinical and clinical curricula.

## Appendices

Appendix A: survey instrument

This appendix contains the full survey (Table 2) created by the authors, approved by the University of Texas Health Science Center at Houston Institutional Review Board (HSC-MS-24-0530), and administered to medical students at McGovern Medical School. The survey included 12 clinical vignettes addressing a range of women’s health-related scenarios. For each vignette, students were asked to select a preferred specialty from a multiple-choice list. In addition, the survey collected demographic information (age and gender), specialty of interest, and prior exposure to obstetrics and gynecology (Ob/Gyn). The purpose of including the complete survey instrument is to provide transparency regarding question design and allow replication in future research.

**Table 2:** Author-developed survey instrument A structured questionnaire was created to assess medical student knowledge of Ob/Gyn. It included two sections: demographics (age, gender, class year, and prior Ob/Gyn exposure via multiple-choice or free response) and clinical vignettes evaluating knowledge of diagnoses and consultation decision-making. “(Free response)” indicates that participants had the opportunity to write in their answers to the demographic questions.

Question	Answer choices
Age	(free response)
Gender	M
	F
	Other: (free response)
	Prefer Not to Answer
Class of	2025
	2026
	2027
	2028
Previous Exposure to Ob/Gyn	Shadowing
	Preceptorship
	Clinical Rotation
	Other: (free response)
	None
Specialty of Interest	(free response)
Who would you refer a patient to for a pap smear?	Family Medicine
	General Surgery
	Ob/Gyn
	Internal medicine
38 year old G2P2 female would like to be permanently sterilized. Who would you refer this patient to for more counseling?	Family Medicine
	Ob/Gyn
	Surgical Oncology
	General Surgery
23 year old G0 female presents to ER with sudden 10/10 LLQ abdominal pain during her soccer game. Who would you consult first?	Trauma surgery
	Urology
	Ob/Gyn
	Internal medicine
34 year old female is experiencing severe abdominal pain and inability to urinate after C-section. Which specialty would you call first?	General Surgery
	Urology
	Vascular Surgery
	Ob/Gyn
54 year old G4P4 female with progressive urinary incontinence while laughing and picking up her grandchildren. Who would you refer her to for definitive treatment?	General Surgery
	Urology
	Primary care
	Ob/Gyn
27 year old G2P1001 at 16 weeks and 2 days is found to have community acquired pneumonia. Which hospital service would you admit her to?	Family Medicine floor
	Ob/Gyn floor
	Internal medicine floor
	Pediatrics floor
16 year old G0 presents with painful cystic acne. Which specialty would you refer her to?	Family Medicine
	Dermatology
	Ob/Gyn
	Internal medicine
35 year old transgender man requires a yearly health maintenance exam. Which specialty would you recommend he see first?	Family Medicine
	Endocrinology
	Ob/Gyn
	Internal medicine
62 year old G3P3 female is found to have ovarian cancer that has metastasized to the para-aortic lymph nodes. Which specialty would you refer her to for a surgical management of her condition?	Ob/Gyn
	Surgical Oncology
	General Surgery
	Urology
37 year old G3P1102 at 12 weeks and 6 days gestation is experiencing chronic knee pain. Which provider would you recommend she see?	Family Medicine
	Orthopedic Surgery
	Ob/Gyn
	Internal medicine
30 year old G2P2 presents with pain while walking, sitting, and during intercourse. Physical exam shows a fluctuant vulvar mass. Who would you consult first?	Ob/Gyn
	Surgical Oncology
	General Surgery
	Urology
40 year old G4P3 at 24 weeks gestation presents with a 1 cm brown and black mole on her face. She says that the mole has enlarged over the past year and would like it removed. Who would you recommend she see to have the mole excised?	Surgical Oncology
	Dermatology
	Plastic Surgery
	Ob/Gyn
